# Unexpectedly low prevalence of hepatitis delta virus infection in Southern Viet Nam

**DOI:** 10.1038/s41598-025-33268-0

**Published:** 2025-12-31

**Authors:** Thuy Nguyen, Van Huy Vo, Long Le, An Bac Luong, Chuong Dinh Nguyen, Phong Tien Quach, Thuy Thi-Thanh Trinh, Sang The Phan, Tuan Ngoc Cao, Thi Bich Chi Mai, Vu Anh Hoang, Hoang Huu Bui, Frank Maldarelli

**Affiliations:** 1 HIV Dynamic and Replication Program, NCI Frederick, NIH, 1050 Boyles Street, Frederick, MD 21702 USA; 2 Department of Gastroenterology, University Medical Center, Viet Nam 217 Hong Bang Street, District 5, Ho Chi Minh, Viet Nam; 3 Center for Molecular Biomedicine, University of Medicine and Pharmacy at Ho Chi Minh, Ho Chi Minh, Viet Nam; 4 Laboratory Department, University Medical Center Ho Chi Minh, 700000 Ho Chi Minh, Viet Nam

**Keywords:** HDV prevalence, Southern viet nam, Standardized assays, Geographic distribution, Diseases, Gastroenterology

## Abstract

Viet Nam faces a significant burden of viral hepatitis-associated liver disease, but the contribution of HDV, the most severe form of viral hepatitis, remains underinvestigated. HDV is substantial in the Northern and Central regions, but has not been documented in the South of Viet Nam. To investigate HDV prevalence and its association with severe liver disease in Southern Viet Nam, we used the standardized assay (LIAISON XL Anti-HDV) to detect HDV antibodies (anti-HDV) in 721 HBsAg positive individuals with hepatitis flare (*n* = 158), liver cirrhosis (LC) (181), hepatocellular carcinoma (HCC) (207), chronic hepatitis B (CHB) (175). Unexpectedly, anti-HDV was rare and only detected in 11/721 participants (1.5%), and not significantly different among groups: 2/158 (1.3%) in flare, 4/181 (2.2%) in LC, and 5/207 (2.4%) in HCC, and 0/175 (0%) in CHB. This suggests that HDV is not one of the major contributors to the high burden of liver disease in Southern Viet Nam. The discrepancy of HDV prevalence between Northern-Central and Southern regions suggests location-specific distribution of HDV, which in turn reflects differences in HDV transmission routes, study populations, and/or study methodologies. Our study underscores the need for tailored, regional screening strategies rather than a single national guideline for HDV infection.

**Supplementary Information:**

The online version contains supplementary material available at 10.1038/s41598-025-33268-0.

## Introduction

Hepatitis delta virus (HDV) is a defective RNA virus that requires the hepatitis B virus (HBV) envelope to infect hepatocytes and establish infection^1^. HDV/HBV coinfection leads to a 2- to 3-fold increase in mortality compared to HBV mono-infection^2^. This co-infection is also a major contributor to severe liver-related complications, including rapidly progressive fibrosis, cirrhosis, and hepatocellular carcinoma (HCC).

Nearly 254 million people were living with chronic hepatitis B (CHB) globally in 2022, resulting in 1.1 million deaths, primarily due to cirrhosis and HCC^3^. Among individuals with CHB, HDV prevalence ranges from 4.5% to 10.6%^4–6^. Some countries have a particularly high burden of HDV infection, especially in Africa and Asia^7^. However, estimation of HDV prevalence varies considerably across regions due to differences in the study population, population mobility, diagnostic methods, and lack of awareness. Testing for HDV in all HBsAg carriers or reflex anti-HDV testing is not consistently recommended by international societies nor widely implemented, even in high-resource settings^8,9^. The European Association for the Study of the Liver recommends screening for HDV in all HBsAg carriers, while the American Association for the Study of the Liver recommends a risk-based screening approach^8,9^. In low-income settings, the implementation of reflex anti-HDV testing is even more challenging due to the lack of evidence on costs and benefits. Therefore, research on HDV prevalence and the characteristics of people living with HDV is essential to provide feasible, scalable, and cost-effective screening approaches.

Viet Nam is a country with high HBV endemicity, with an estimated 8.1% prevalence among the general population, posing a growing public health burden^10^. The country ranks fourth globally in the incidence of HCC, a burden attributed to multiple risk factors such as HBV, hepatitis C virus (HCV) infections, increased alcohol use, and metabolic liver diseases^11,12^. Although the contribution of HDV to HCC in Viet Nam is not well understood, it is likely to be significant. A recent meta-analysis reported an HDV antibody prevalence of 7.93% and an HDV RNA positivity rate of 13.9% in Viet Nam^13^. Variation in positive rates of HDV antibody and HDV RNA may reflect heterogeneity in the study populations, variability in assay sensitivity and specificity, and, more importantly, non-standardized research methodologies because most studies only performed either anti-HDV or in-house HDV-RNA assays to detect HDV infection.

HDV infection in Viet Nam is concentrated in individuals at high likelihood of HDV infection or those with acute hepatitis B, cirrhosis, and HCC^13,14^. However, most studies were conducted in the Northern and Central regions, leaving a critical gap in Southern Viet Nam, which includes Ho Chi Minh City—the nation’s most populous urban center. Given the geographically heterogeneous distribution of HDV in other settings, expanded research in Southern Viet Nam is necessary. To address this gap, we estimated the prevalence of HDV infection in Southern Viet Nam among CHB participants with distinct liver manifestations (hepatitis flares, cirrhosis, HCC, and stable CHB), using standardized assays approved for the clinical management of HDV. This single-site cross-sectional study of over 700 CHB participants provides critical epidemiological data and offers essential insights to guide clinical management and public health strategies.

## Methods

### Study cohort

This cross-sectional study was conducted at a single site—the University Medical Center in Ho Chi Minh City—between April 2023 and March 2025. The center is a major tertiary referral hospital, providing care for patients from various southern provinces of Viet Nam. HBsAg-positive individuals at different stages of liver disease were enrolled, including participants presenting with acute or recent (within the past 3 years) hepatitis flare (flare), cirrhosis (LC), hepatocellular carcinoma (HCC), and stable chronic hepatitis B (CHB). Participants were diagnosed as having a hepatitis flare if their Alanine aminotransferase (ALT) was greater than 5 times the upper limit of normal (ULN) (ULN for men: 40 U/L, and for women: 31 U/L). Cirrhotic participants were diagnosed by physical examinations, such as clinical manifestation of hepatic failure syndrome or portal hypertension, combined with imaging such as transient elastography or fibroscan (cut-off 12 kPa), and laboratory analyses such as the fibrosis index based on four factors or aspartate transaminase-to-platelet ratio index. The diagnosis for HCC was established based on the results of dynamic multiphasic contrast-enhanced computed tomography, magnetic resonance imaging, and tumor biomarkers (AFP, AFP-L3, PIVK-II) (HCC group). Participants with transaminase levels lower than 2 ULN were enrolled in the chronic hepatitis B group. Participants with active intercurrent illness, infection, co-infection with HCV, or having hepatitis due to alcohol addiction, drug toxicity, or pregnancy were excluded. Epidemiological including age, sex, place of residence, and liver-related clinical data (qualitative results of HBsAg, HbeAg, hematological and biochemical parameters of liver function, degree of liver fibrosis, HBV DNA if available, and anti-HBV drug regimen) were collected from the medical records.

### Measurement of total HDV antibodies

The presence of total HDV antibodies (anti-HDV) was measured using an assay approved for in vitro diagnostics (LIAISON XL Anti-HDV)^15^. The assay was performed using 170 µL of serum/plasma on the LIAISON platform (Mitalab, Ho Chi Minh City, Viet Nam) per manufacturer’s protocol.

### Viral nucleic acid isolation

Viral nucleic acids were extracted from 400 µL plasma/serum of anti-HDV positive samples and eluted in 60 µL water using the Quick-DNA/RNA Viral Kit (Zymo, USA, Cat#D7020) following the manufacturer’s protocol.

### HDV-RNA quantification

HDV RNA levels were measured using the research version of a conformité européenne IVD (CE-IVD)-labeled assay RoboGene HDV RNA Quantification Kit 2.0 (Roboscreen, Germany, Cat#847–0207400584-RUO), following the manufacturer’s protocol. Briefly, for each sample reaction, 5 µL of extracted viral nucleic acids was combined with 20 µL of master mix containing 6.25 µL RT PCR Enzyme Mix FS, 1 µL 25x Reagent Mix, and 12.75 µL of PCR-grade water. The ready-to-use standards, calibrated against the 1 st WHO HDV RNA reference material, were prepared with the same reaction components. Amplification was performed on the LightCycler Roche 480 II using the following conditions: reverse transcription at 55 °C for 15 min, Taq activation at 95 °C for 2 min, and 45 cycles of denaturation at 95 °C for 15 s and annealing/elongation at 60 °C for 1 min, followed by cooling at 40 °C for 30 min. This assay allows highly sensitive detection of HDV RNA in all eight genotypes. The limit of detection (LoD) of the assay on the LightCycler Roche 480 is 14 international units (IU)/mL, and the quantification linear range is 5 to 1 × 10^8^ IU/mL.

### HDV genotyping

Eight µL of extracted viral nucleic acid were denatured at 94 °C for 3 min, followed by a snap freezing step in −80°C, and reverse transcribed with the SuperScript IV system (Invitrogen, Life Technologies, USA, Cat# 18090050) and 0.1 µM HDV-specific reverse primer (HDV-878R: ATGCCCAGGTCGGACCGCGAGGA) as previously described^16^. HDV cDNA was 2-fold diluted, and amplified using the Platinum II Taq Polymerase system with forward primer (HDV-304 F: CTCCAGAGGACCCCTTCAGCGAAC) and reverse primer (HDV-1264R: CTTGTTCTCGAGGGCCTTCCTTCG) at 1 µM final concentration in a 20 ul reaction volume. PCR was carried out with the following cycling program: 94 °C for 2 min, 40 cycles of 94 °C for 15 s, 60 °C for 15 s, and 68 °C for 30 s, and one cycle of final extension at 68 °C for 5 min. The semi-nested PCR was performed with the same enzyme system and cycling conditions using the forward primer (HDV-466 F: GAGTGAGGCTTATCCCGGGG) and reverse primer (HDV-1264R: CTTGTTCTCGAGGGCCTTCCTTCG). Amplicon bands of 798 bp were checked on gel electrophoresis and purified using the NucleoFast PCR ultrafiltration kit for PCR clean up (Machery Nagel, USA, Cat#743500.4) and submitted for Sanger sequencing (Psomagen, Rockville, USA).

### HBV-DNA quantification

For HBV DNA values collected from the medical records, HBV DNA was measured by using either the automatic Roche Cobas 4800 system with a linear range of 10 IU/mL to 1.0E + 09 IU/mL (KIT COBAS 4800 HBV 120 T CE-IVD, Roche, Germany, cat#06979564190) or the HBV Real-TM Quant Dx system (Amerigo Scientific, USA, cat# PK01090225SAC) with a linear range of 7 IU/mL to 1.0E + 08 IU/mL. For anti-HDV positive samples, we quantified HBV-DNA using digital droplet PCR to increase the detection sensitivity. Briefly, four ul of viral nucleic acid was combined with 10 ul of 2X ddPCR SuperMix for Probes (Bio-Rad Laboratories, USA, Cat#1863024), 0.75 µM forward primer (HBVX-F1779: GGCTGTAGGCATAAATTGG), 0.75 µM reverse primer (ACAGCTTGGAGGCTTGAA), and 0.25 µM of probe (HEX-TTCACCTCTGCCTAATCATCTCATGT-NFQ-MGB) at a final concentration and nuclease-free water to a total volume of 20 µl. Droplets containing the duplicate of the reaction mixture were run on a thermocycler with the following program: initial denaturation at 95 °C for 10 min, followed by 45 cycles of 94 °C for 30 s and 59 °C for 1 min with a 2 °C/s ramp rate, and a final enzyme deactivation step at 98 °C for 10 min. Droplets were analyzed using the QX200 Droplet Reader (Bio-Rad) with QuantaSoft software (Bio-Rad, version 1.7.4). The LOD of the technique is 7.5 copies/mL plasma.

### HBV genotyping

In brief, 2.5 ul of extracted viral nucleic acids was amplified by PCR using the Platinum II Taq Hot-Start DNA Polymerase system (ThermoFisher, USA, Cat#14966025) following the manufacturer’s protocol with 0.2 µM forward primer (P1-1821 F: TTTTTCACCTCTGCCTAATCA) and 0.2 µM reverse primer (P2-1826R: AAAAAGTTGCATGGTGCTGG) at final concentrations in a 10 µL reaction^17^. First-round PCR products were diluted 5-fold and were submitted for a second round of PCR using the same enzyme system with 0.2 µM forward primer (BCPF-1852 N: ATGTCCTACTGTTCAAGCCTC) and 0.2 µM reverse primer (MDN5R-1775 N: ATTTATGCCTACAGCCTCCT)^18^. HBV amplicons were checked on gel electrophoresis and purified using NucleoFast 96 PCR ultrafiltration kit for PCR clean up (Machery Nagel, USA, Cat#743500.4) and submitted for Sanger sequencing with three primers (BCPF-1852 N, MDN5R-1775 N, and POL-2814+: GGGTCACCATATTCTTGGGAAC) (Psomagen, Rockville, MD, USA). A region of 200 bp from position 635–835 (referenced to LC064381) was missed in some sequences due to a lack of sequencing coverage.

### Sequencing data analysis

Low-quality sequences were discarded, and trimmed sequences were aligned to the HBV-B reference derived from a Vietnamese individual (accession# LC064381) or HDV reference (HDV X04451.1) in Genious Prime v 2025.1.2 (https://www.geneious.com). HBV genotyping was performed with the NCBI HBV genotyping tool, and HDV genotypes were identified through the NCBI Blastn tool. Approximate maximum-likelihood trees were inferred using Fast Tree 2.1 with the Generalized Time-Reversible model and the Shimodaira-Hasegawa test for local support values^19^. Clusters of sequences with genetic distance < 2% and branch supports > 0.9 were identified using ClusterPicker (https://hiv.bio.ed.ac.uk/software.html)^20^.

### Statistics analysis

Statistical analyses were performed in GraphPad Prism version 10. The unpaired Mann-Whitney test was performed to compare participant characteristics between groups. Fisher’s exact test was used to compare HDV prevalence between the severe liver disease and CHB groups.

### Ethics

The study was approved by the Ethical Review Board at the University of Medicine and Pharmacy at Ho Chi Minh City, Viet Nam (Protocol IRB-VN01002/IORG0008603/FWA00023448). All participants provided informed consent and agreed for their remnant blood samples to be used for this research. All methods were performed in accordance with the relevant guidelines and regulations of the institutions.

## Results

### Participant characteristics

We enrolled 721 HBsAg-positive participants with distinct clinical manifestations, including 158 flare, 181 LC, 207 HCC, and 175 CHB participants. Participants’ characteristics were summarized in Table 1. Briefly, the median (interquartile range-IQR) ages of participants in the flare, LC, HCC, and CHB groups were 44 (37–53), 56 (49–66), 61 (54–68), and 47 (40–56) years, respectively. HCC and LC participants were significantly older than flare and CHB participants (adjusted p-values = < 0.0001–0.0014). Men accounted for more than 50% in all groups and were significantly more predominant in the flare and HCC groups (77.2% in flare, 82.1% in HCC vs. 52.0% in CHB and 60.7% in LC) (p-values < 0.0001). Compared to other groups, flare participants had significantly higher levels of transaminase, with the median (IQR) of ALT of 577 (336–1248 U/L) and AST levels of 397 (221–826) U/L at enrollment (p-values < 0.0001). They also had a significantly higher proportion of positive HBeAg (45.0%) and HBV-DNA levels (median (IQR) of 6.45 (4.03–7.76) log (IU/mL)) (p-values < 0.0001). A majority of participants (70.3%) experienced a spontaneous flare, while 22.2% experienced a flare due to nucleos(t)ide analogues (NA) withdrawal. Two participants who experienced flares also had cirrhosis and were classified into the flare group. All flare participants were off NA treatment at the time of flare, with a median (IQR) of HBV DNA of 6.45 (4.07–7.76) log (IU/mL), but all initiated NA treatment immediately after flare diagnosis. All LC and HCC participants were treated and had medians (IQR) of HBV DNA levels of 1.30 (1.30–3.21) and 1.30 (1.30–4.55) log (IU/mL), respectively. In the CHB group, 84/175 (48%) participants were treated with a median HBV DNA level (IQR) of 2.22 (1.30–3.88) log (IU/mL) for all participants.

**Table 1:** Participant characteristics.

Characteristic Median (Interquartile range)	Flare (*n* = 158)	LC (*n* = 181)	HCC (*n* = 207)	CHB (*n* = 175)	*p*-value
Age (years)	44 (37–53)	56 (49–66)	61 (54–68)	47 (40–56)	< 0.0001
Female/male (%/%)	36/122 22.8/77.2	71/110 39.3/60.7	40/167 17.9/82.1	84/91 48/52.0	< 0.0001#
ALT (U/L)	577 (336–1248)	34 (24–55)	36 (24–52)	23 (17–31)	< 0.0001
AST (U/L)	397 (221–826)	45 (34–65)	47 (31–77)	27 (23–31)	< 0.0001
GGT (U/L)	192 (119–314)	58 (32–96)	97 (50–226)	23 (17–36)	< 0.0001
Albumin (g/L)	38.4 (32.9–41.7)	40.2 (35.1–44.2)	41.0 (35.4–43.3)	NA	< 0.0001
Total Bilirubin (mg/dL)	3.43 (1.07–18.66)	1.14 (0.82–1.67)	0.86 (0.67–1.35)	NA	< 0.0001
Platelets (K/uL)	204 (155–260)	117 (80–143)	174 (127–245)	240.5 (206–285)	< 0.0001
Positive qualitative HBeAg (%)	45.0	21.3	26.7	20.0	< 0.0001#
HBV-DNA (log IU/mL)*	6.7 (3.97–7.97)	2.21 (1.23–4.54)	2.41 (1.00–4.81.00.81)	3.45 (2.18–5.33)	< 0.0001

ALT: Alanine aminotransferase, AST: Aspartate aminotransferase, GGT: gamma-glutamyl transferase, HBeAg: Hepatitis B e-antigen. *: HBV-DNA measurements in all groups were collected from the medical records at or within 3 months of sampling. Undetectable HBV-DNA levels were assigned to the lower limit of quantification of 10 IU/mL or 1.00 log HBV-DNA (IU/mL). #: Features were compared by the Chi-square test. Other features were compared by the Kruskal-Wallis test.

### HDV infection did not contribute to the burden of liver disease in Southern Viet Nam

HDV is not the major contributor to the burden of liver disease in our study population. Overall, anti-HDV were present in 11/721 (1.5%) participants. Across groups, anti-HDV were found in 2/158 (1.27%) participants in Flare, 4/181 (2.21%) in LC, 5/207 (2.42%) in HCC, and 0% (0/175) in CHB groups. HDV infection was more frequently observed in participants with severe liver disease, including flare, LC, and HCC (2.01%) compared to stable CHB participants (0%), but the difference was not statistically significant (*p* = 0.0747) (Table 2).

**Table 2:** HDV infection in participants with severe liver disease and stable CHB.

	Severe Liver Disease (Flare, LC, HCC)	CHB	*P*-value Fisher’s exact test
Positive anti-HDV (n)	11	0	0.0747
Negative anti-HDV (N)	535	175	

**Fig. 1:**
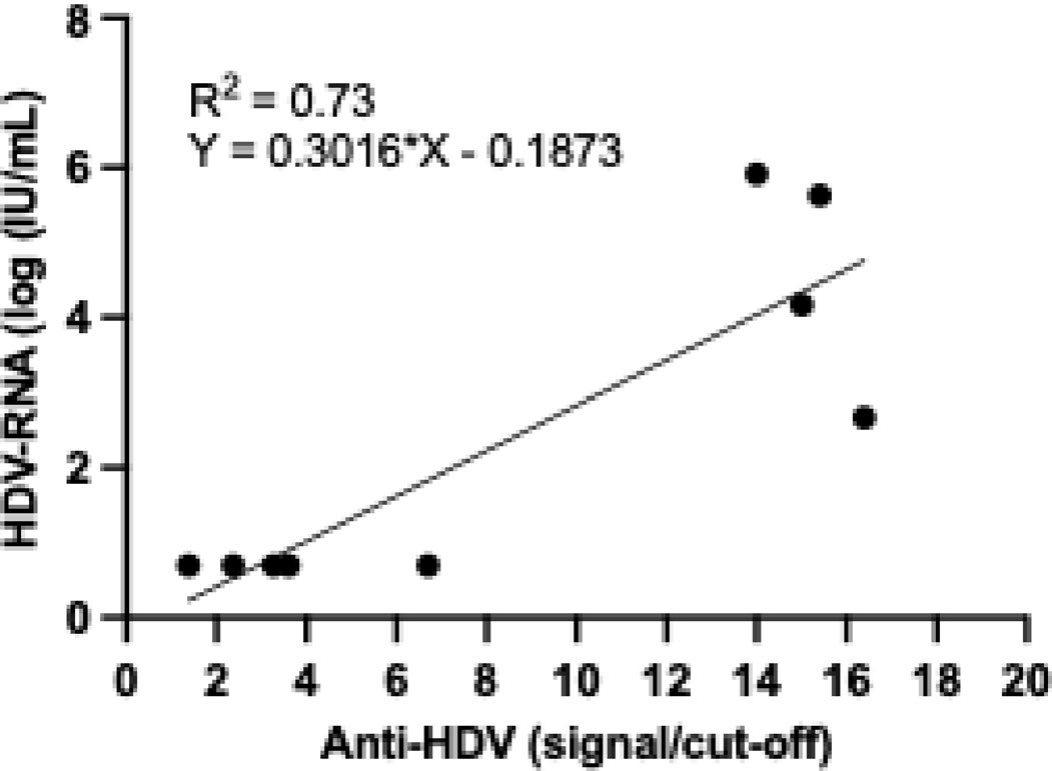
Correlation of anti-HDV (signal/cut-off) and HDV-RNA levels (log(IU/mL)).

HDV-RNA was measured in 9/11 anti-HDV positive participants and found positive in 4/9 (44.4%) participants, including 1/2 flare, 1/3 LC, and 2/4 HCC participants. Interestingly, we observed a significant association of anti-HDV level (signal/cut-off ratio) and HDV-RNA levels (p-value = 0.03, Spearman correlation coefficient *r* = 0.75) (Fig. 1). The characteristics of 11 participants with positive anti-HDV are listed in Table 3.

**Table 3:** Characteristics of participants with positive HDV antibodies.

Participant ID	Group	Age (years)	Sex	HBV-DNA (copies/mL)	Anti-HDV level (Signal/Cut-off)	HDV-RNA (IU/mL)
1	Flare + LC	56	M	UD	3.61	UD
2	Flare + LC	56	F	UD	15.00	1.48E + 04
3	LC	65	F	UD	6.71	UD
4	LC	32	M	45.0	16.40	4.66E + 02
5	LC	51	M	22.5	2.37	UD
6	LC	56	F	UD	1.26	NA
7	HCC	56	F	UD	14.00	8.29E + 05
8	HCC	76	M	22.5	3.27	UD
9	HCC	52	M	UD	1.38	UD
10	HCC	69	F	UD	15.40	4.26E + 05
11	HCC	57	M	UD	1.59	NA

UD: under the detection threshold. The limit of detection for HDV-RNA was 5 IU/mL, and for HBV-DNA was 7.5 copies/mL by ddPCR. NA: samples not available for HDV quantification.

### Specificity of HDV strains Circulating in Viet Nam

**Fig. 2:**
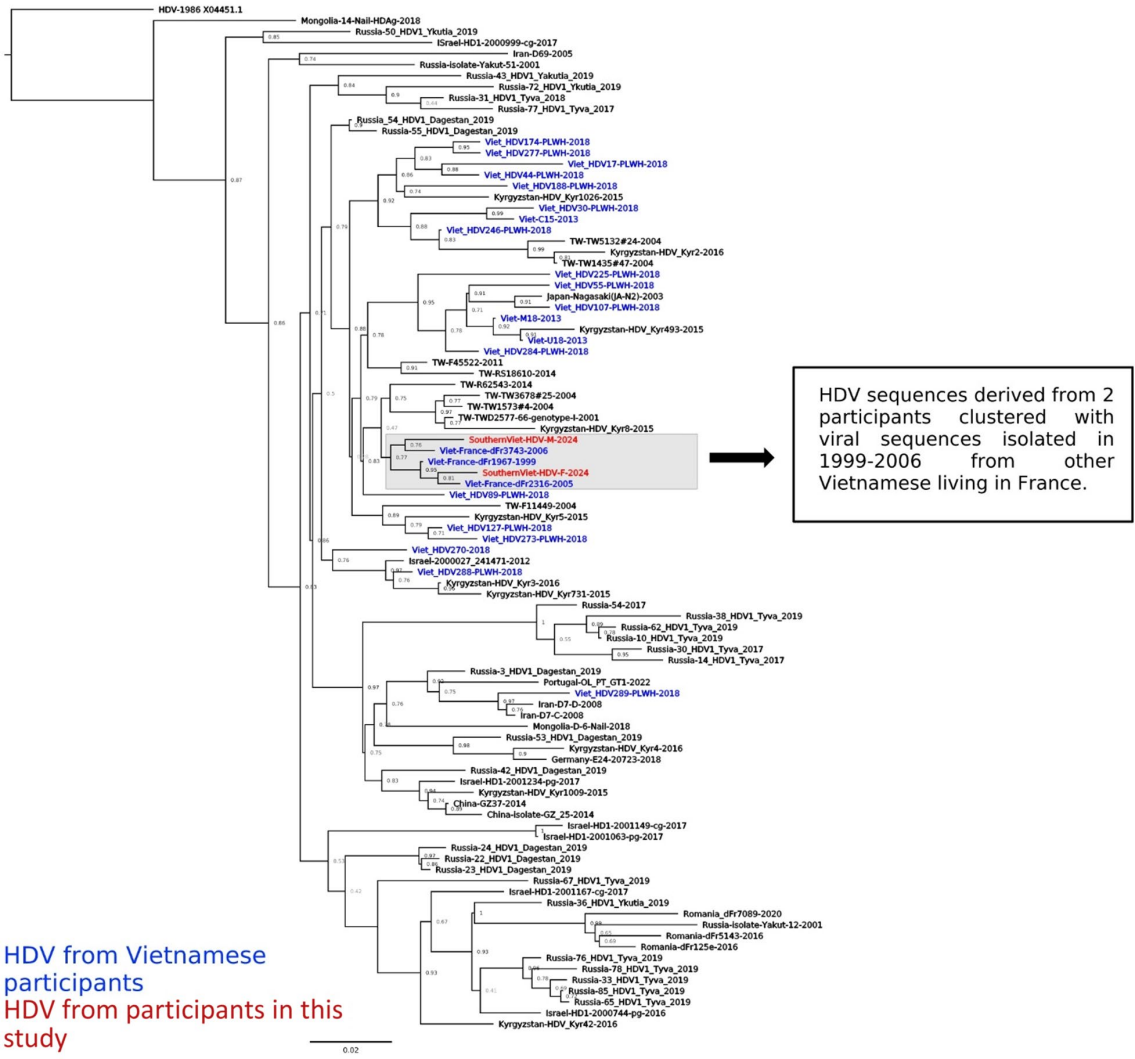
Phylogenetic tree of HDV sequences (575 bp, positions 500–1055 to the reference HDV X04451.1) from participants in this study with the most similar HDV sequences from the literature (> 93% of identity). Sequences were annotated with the country and the year in which the study was conducted. HDV sequences derived from Vietnamese people were highlighted in blue, and those from this study were highlighted in red.

Among the four participants with positive HDV-RNA, we successfully sequenced a 700 bp fragment of the HDV large antigen region from two participants with HDV-RNA > 10E5 IU/mL (participants 7 and 10) who were found infected with HDV genotype 1. Their sequences were blasted against the GenBank database to identify the most similar (> 93% identity) HDV sequences in the literature. Interestingly, HDV isolated from the two participants in this study established a cluster with viruses isolated from 1999 to 2006 from other Vietnamese living in France (Fig. 2). Furthermore, HDV was found to be clustered among Vietnamese people only, regardless of residency locations.

### HBV genotype distribution

We performed sequencing of the full-length HBV for genotyping and genetic characterization on 55 participants with measurable HBV DNA. Among them, 25 had Flare, 7 had CHB, 8 had HCC, and 15 had LC. We found that 44/55 (80.00%) of participants were living with HBV genotype B (HBV-B), and 11/55 (20.00%) were living with HBV genotype C. Interestingly, we identified a distinct and large cluster of HBV-B isolated from a majority of HCC (*n* = 7) and LC (*n* = 10) participants (Supplemetary data, Fig. S1). The average pairwise distance (APD), a measure of viral diversity, of viruses in this cluster was low at 0.78% (subs/site), indicating the high similarity of viruses responsible for HCC and LC development in these participants. Furthermore, this cluster of viruses exhibited distinct patterns of nucleotide variations (Supplementary data, Table S1) across the entire viral genome, particularly the G1613A mutation, which is known to be associated with HCC development.

## Discussion

Viet Nam faces a significant burden of HBV infection and HBV-associated liver disease, including liver cancer, with more than twenty-four thousand new cases a year^21^. HDV, a satellite virus of HBV, can be the reason contributing to this high burden of liver cancer in Viet Nam. In Viet Nam, HDV is surveyed mostly in the Northern and Central areas, showing the overall prevalence of 7.93% for HDV antibodies, while it is 13.9% for positive HDV-RNA^13^. The diagnostic accuracy of serological tests for HDV varies widely, with sensitivities from 51.9% to 95.3% and specificities from 80% to 95.3%^22^. The accuracy of HDV-RNA measurement also differs significantly across commercial and in-house assays, with several log_10_ differences due to experimental procedures, HDV diversity, and secondary structure^23–26^. In this study, we investigated the prevalence of HDV using standardized techniques with proven excellent performance in the diagnosis and clinical management of HDV infection^15,27^.

Besides the strong association with cirrhosis and liver cancer, HDV infection can also lead to hepatitis flare if superinfected in HBsAg carriers^28^. The contribution of HDV to hepatitis flare has never been investigated in Viet Nam, nor the surrounding countries. Given the severity of HDV-associated liver disease, we investigated the HDV infection rate in 721 HBsAg carriers, including 158 hepatitis flare, 181 LC, 207 HCC, and 175 CHB participants. HDV was rare and detected only in 11/721 participants in our study population. HDV was found in 1.27% of flare, 2.21% of LC, 2.42% of HCC, and 0% of CHB participants. While HDV infection was more frequently observed in participants with severe liver disease, including flare, LC, and HCC (2.01%) compared to stable CHB participants (0%), the difference was not statistically significant, which is likely due to the low HDV prevalence. This indicated that HDV is not the major contributor to the burden of liver disease in our study population. This result also revealed the interesting geographic discordance of HDV distribution within the country, where the Northern and Central areas have a higher prevalence of HDV, ranging from 8 to 14% overall and up to 14–15% in participants with cirrhosis or HCC^13^. This suggests potential differences in HDV transmission routes, characteristics of people at risk for HDV infection, or study methodologies. Further studies using the same methods, including study design, study populations, and standardized HDV diagnosis assays, are necessary to thoroughly investigate this discrepancy.

Among positive anti-HDV participants, 44% have positive HDV-RNA, indicating the active replication of the virus and the importance of close monitoring using HDV-RNA for the progression of liver disease. This finding is similar to other studies suggesting the role of HDV RNA testing in the clinical management of HDV infection^26^. Interestingly, we observed a significant association of HDV RNA level with HDV antibody titers despite the qualitative nature of anti-HDV measurement. This suggests the use of anti-HDV titers for initial assessment of HDV-RNA level if the assay is unavailable or not affordable. However, the use of HDV-RNA is irreplaceable to assess viral response to antiviral treatment or monitor viral replication. Therefore, affordable or rapid HDV-RNA testing is still required in resource-limited countries.

Positive HDV-RNA samples were subjected to sequencing for genotyping and genetic characterization. The two participants with successful sequencing showed infection of HDV genotype 1, the most dominant viral genotype in Viet Nam. Interestingly, phylogenetic analysis showed that HDV sequences isolated from participants in this study are clustered with viruses isolated in 1999 and 2006 from Vietnamese people living in France. This suggests the long-term establishment of HDV infection and also the lack of viral evolution. The cluster of HDV from only Vietnamese people indicates the specificity of HDV strains circulating in Viet Nam, suggesting that the transmission occurs domestically rather than at an inter-country level.

Our study has some limitations. This is a cross-sectional study with more than 700 participants attending only one clinical center. Therefore, the HDV infection rate may not reflect the prevalence of HDV across the southern region. However, the center is a referral hospital from which participants can come from both Ho Chi Minh City and other southern provinces. This suggests that our study population still likely reflects the geographic distribution of HDV among HBsAg carriers linked to care in Southern Viet Nam. However, it is worth noting that a majority of people living with HBV in Viet Nam are not linked to care due to a lack of disease awareness; therefore, the rate of HDV infection found in this study does not reflect the prevalence of HDV in the entire Southern region^29^. Another limitation is that we do not know the risk factors for HDV acquisition in our study participants. HDV infection is more concentrated in people who have a higher likelihood of HDV acquisition, such as those who inject drugs or men who have sex with men. The difference in risk factors between our study participants and other studies may partially explain the difference in HDV prevalence between the Northern-Central and Southern areas.

In conclusion, our study is the first study in Viet Nam using standardized assays for the diagnosis and clinical management of HDV infection. We found an unexpectedly low rate of HDV infection, indicating that HDV infection is not a major contributor to the high burden of severe liver disease in Southern Viet Nam. The discrepancy of HDV prevalence between regions suggests location-specific distribution of HDV, which in turn reflects differences in HDV transmission routes, study populations, and/or study methodologies. This discrepancy underscores the need for tailored, regional screening strategies rather than a single national guideline.

## Supplementary Information

Below is the link to the electronic supplementary material.

Supplementary Material 1

## Data Availability

The data that support the findings of this study are available upon request from the corresponding author. Sequencing data generated during the current study are available at GenBank with the accession numbers PX440300-PX440354 and PX393450-PX393451.

## Declarations

### Meetings

The study was presented at the Asian Pacific Digestive Week in November 2024 and Asian Pacific Association for the Study of Liver (APASL) in March 2025.
