# Supplementary material for: Unexpectedly low prevalence of hepatitis delta virus infection in Southern Viet Nam
Source: Sci Rep. 2025 Dec 31;16:3307. doi: 10.1038/s41598-025-33268-0 (PMC12835169; doi:10.1038/s41598-025-33268-0)
Supplement: Supplementary file 1 — Supplementary Material 1 [file 41598_2025_33268_MOESM1_ESM.docx]

**Unexpectedly Low Prevalence of Hepatitis Delta Virus Infection in Southern Viet Nam**

Thuy Nguyen^1*^,  Van Huy Vo^2^,  Long Le^1^, An Bac Luong^3^,  Chuong Dinh Nguyen^2^,  Phong Tien Quach^2^,  Thuy Thi-Thanh Trinh^2^,  Sang The Phan^2^, Tuan Ngoc Cao^2^, Thi Bich Chi Mai^4^,  Vu Anh Hoang^3^,  Hoang Huu Bui^2*#^, Frank Maldarelli^1#^

^1^ HIV Dynamics and Replication Program, NCI, Frederick, United States,

^2^ Department of Gastroenterology, University Medical Center, Ho Chi Minh City, Viet Nam,

^3^ Center for Molecular Biomedicine, University of Medicine and Pharmacy at Ho Chi Minh City, Viet Nam,

^4^ Laboratory Department, University Medical Center Ho Chi Minh City, Ho Chi Minh City 700000, Viet Nam

#: These authors contributed equally to this work.

**S1:** **Identification of a large cluster of HBV-B derived from HCC and LCC participants**

**
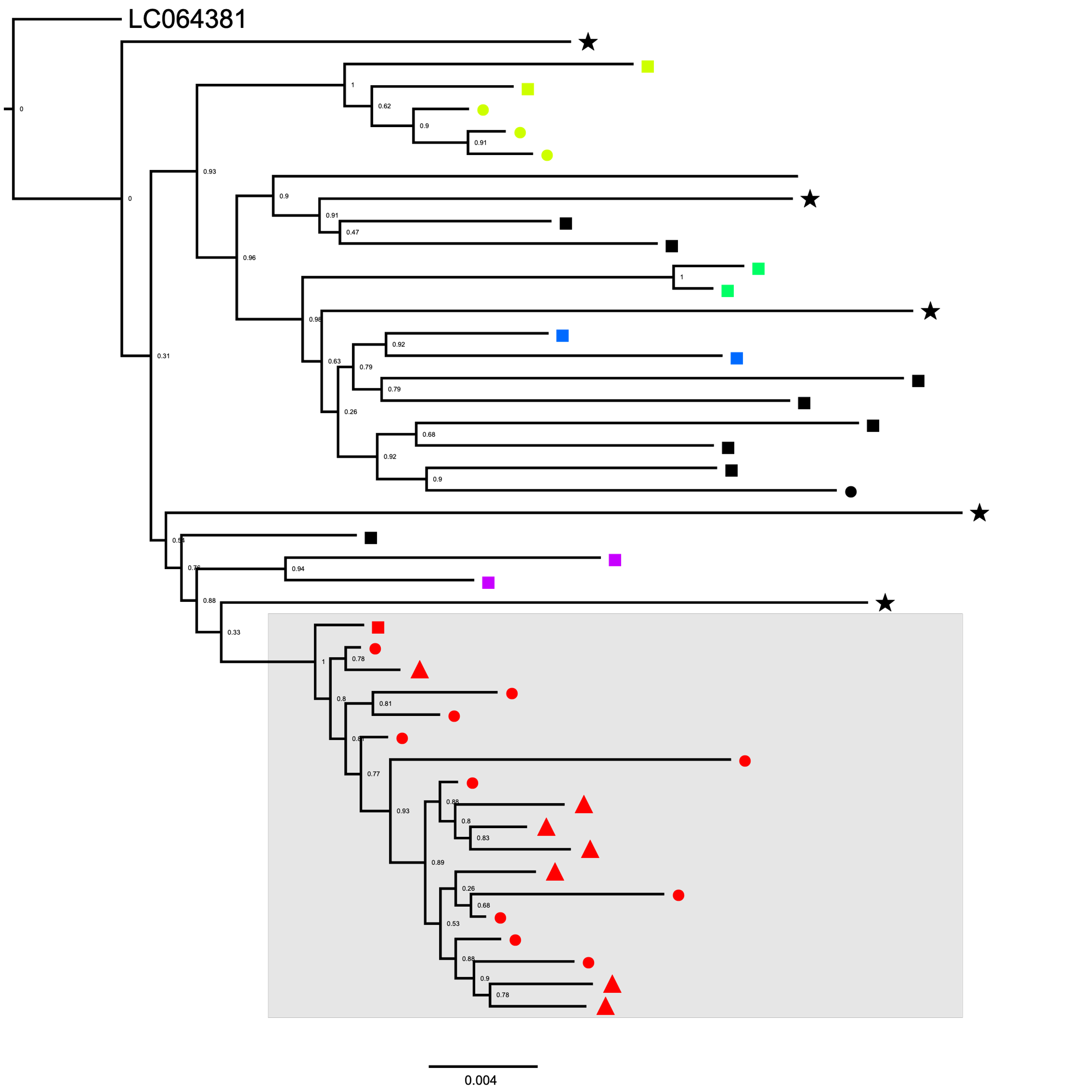
**

■: Flare, ●: LC,

▲: HCC, ⋆: CHB

**Figure S1: Phylogenetic tree of HBV genotype B (HBV-B) sequences from all participants.**

Viral clusters were identified and denoted in the same color via ClusterPicker using the threshold of 2% genetic distance and 0.9 bootstrap support values. The largest cluster (highlighted in red) consisted of a majority of sequences from HCC (n=7) and LC (n=10) participants.

**S2: Distinct nucleotide variations of viruses derived from HCC and LC participants**

Sequences of viruses within the largest cluster (N=18) were compared with sequences outside this cluster (N=26) at nucleotide positions. We reported positions that showed a more than 2-fold difference in nucleotide frequency between viruses inside and outside the largest cluster. Nucleotide positions were referenced to the HBV reference sequence derived from a Vietnamese isolate (GeneBank accession # LC064381).

**Table S1: Distinct allele frequency between HBV-B inside and outside the large cluster**

| LC064381 nucleotide-position | In-cluster frequency (%) (N=18) | Out-cluster frequency (%)  (N=26) | LC064381 nucleotide-position | In-cluster frequency (%) (N=18) | Out-cluster frequency (%) (N=26) |
| --- | --- | --- | --- | --- | --- |
| G355 | G (94.4), A (5.6) | G (12.5), A (87.5) | T1990 | T (0), C (100) | T (65.4), C (30.8) |
| A551 | A (5.6), T (94.4) | A (96.2), T (0) | C2073 | C (33.3), G (61.1) | C (96.4), G (0) |
| T777 | T (5.6), C (83.3) | T (61.5), C (3.85) | G2088 | G (38.9), T (55.6) | G (88.5), T (7.7) |
| A895 | A (0), G (100) | A (80.8), G (15.4) | C2158 | C (38.9), A (55.6) | C (100), A (0) |
| A933 | A (5.6), G (94.4) | A (100), G (0) | C2304 | C (0), A (100) | C (57.7), A (34.6) |
| C941 | C (100), T (0) | C (50), T (50) | G2357 | G (33.3), T (61.1) | G (80.8), T (7.7) |
| G1062 | G (100), A (0) | G (26.9), A (73.1) | G2386 | G (0), A (94.4) | G (57.7), A (38.5) |
| A1173 | A (100), G (0) | A (50.0), G (50.0) | A2507 | A (5.6), T (94.4) | A (92.3), T (0) |
| G1182 | G (5.6), A (94.4) | G (100), A (0) | A2607 | A (100), G (0) | A (30.8), G (57.7) |
| C1350 | C (5.6), A (94.4) | C (73.1), A (19.2) | C2940 | C (5.6), T (94.4) | C (84.6), T (0) |
| G1368 | G (5.6), A (94.4) | G (80.8), A (3.8) | A2999 | A (0), C (100) | A (92.3), C (7.7) |
| C1494 | C (94.4), T (5.6) | C (50.0), T (50.0) | C3040 | C (0), T (100) | C (94.4), T (5.6) |
| G1613 | G (0), A (94.4) | G (88.5), A (11.5) | A3049 | A (0), G (100) | A (84.6), G (0) |
| ATA1641-1643 | ATA (100),  CCC,CCA,ACC,ACA (0) | ATA (0)  CCC,CCA,ACC,ACA (100) | A3050 | T (100), C (0) | T (30.8), C (50) |
| C1645 | C (100), A (0) | C (26.7), A (61.5) | C3150 | C (100), A (0) | C (30.8), A (57.7) |

**References**

(1) Bui, T. T. T.; Tran, T. T.; Nghiem, M. N.; Rahman, P.; Tran, T. T. T.; Dinh, M. N. H.; Le, M. H.; Nguyen, V. V. C.; Thwaites, G.; Rahman, M. Molecular Characterization of Hepatitis B Virus in Vietnam. *BMC Infect Dis* **2017**, *17* (1), 601. https://doi.org/10.1186/s12879-017-2697-x.

(2) Fang, Z.-L.; Hué, S.; Sabin, C. A.; Li, G.-J.; Yang, J.-Y.; Chen, Q.-Y.; Fang, K.-X.; Huang, J.; Wang, X.-Y.; Harrison, T. J. A Complex Hepatitis B Virus (X/C) Recombinant Is Common in Long An County, Guangxi and May Have Originated in Southern China. *J Gen Virol* **2011**, *92* (Pt 2), 402–411. https://doi.org/10.1099/vir.0.026666-0.
